# Chemical chaperone 4-phenylbutyrate prevents endoplasmic reticulum stress induced by T17M rhodopsin

**DOI:** 10.1186/2045-3701-4-75

**Published:** 2014-12-04

**Authors:** Haibo Jiang, Siqi Xiong, Xiaobo Xia

**Affiliations:** Department of Ophthalmology, Xiangya Hospital, Central South University, 87 Xiangya Road, Changsha, Hunan 410078 P.R. China

**Keywords:** Retinitis pigmentosa, Rhodopsin, UPR, Misfolded, ERAD

## Abstract

**Background:**

Rhodopsin mutations are associated with the autosomal dominant form of retinitis pigmentosa. T17M mutation in rhodopsin predisposes cells to endoplasmic reticulum (ER) stress and induces cell death. This study aimed to examine whether chemical chaperone 4-phenylbutyrate prevents ER stress induced by rhodopsin T17M.

**Results:**

ARPE-19 cells were transfected with myc-tagged wild-type (WT) and T17M rhodopsin constructs. Turnover of WT and T17M rhodopsin was measured by cycloheximide chase analysis. The activity of ubiquitin-proteasome system was evaluated by GFPU reporter. We found that T17M rhodopsin was misfolded, ubiqutinated and eliminated by ER-associated degradation pathway (ERAD) in ARPE-19 cells. Accumulated T17M rhodopsin induced unfolded protein response, but had no effect on the activity of ubiquitin proteasome system. Moreover, chemical chaperone 4-phenylbutyrate facilitated the turnover of T17M rhodopsin and prevented apoptosis and ER stress induced by T17M rhodopsin.

**Conclusions:**

Chemical chaperone could attenuate UPR signaling and ER stress induced by T17M rhodopsin and has potential therapeutic significance for retinitis pigmentosa.

## Background

Retinitis pigmentosa (RP) is considered the most commonly inherited retinal dystrophy with an estimated prevalence of approximately 1:4000 [1]. RP is caused by the progressive loss of rod and cone photoreceptors with clinical hallmarks including the sensitivity to dim light, abnormal visual function and characteristic bone spicule deposits of pigment in the retina [2]. Mutations in rhodopsin, a photon receptor that initiates phototransduction, have been linked to autosomal dominant retinitis pigmentosa (ADRP), accounting for about 10% of all reported cases of RP [3, 4]. Since the identification of the P23H mutation, more than 130 different mutations of rhodopsin have been shown to cause RP [5].

A mouse model of ADRP was created with a threonine-to-methionine mutation at the 17th residue of rhodopsin, which abolishes the glycosylation site at Asn15 and results in a class I RP phenotype [6, 7]. Transgenic mice carrying human T17M rhodopsin gene showed significant photoreceptor apoptosis as early as 24 h after illumination, while mice expressing a rhodopsin transgene with P23H mutation were only minimally affected [8]. Further study showed that endoplasmic reticulum (ER) stress response is involved in retinal degeneration in T17M rhodopsin retinas *in vivo*, accompanied by the up-regulation of autophagy markers and the activation of mitochondrial apoptosis via the up-regulation of pro-apoptotic Bcl2 [9]. Our previous study showed that T17M rhodopsin accumulated in ER, increased the cytotoxicity and predisposed the cells to ER stress induced cell death [10].

Misfolded proteins that do not pass ER quality control (ERQC) are selectively recognized and cleared during a process called ER-associated degradation (ERAD) that involves the export of misfolded proteins from ER followed by proteasomal degradation [11]. Up to now, the role of ERAD in the clearance of T17M rhodopsin is unclear. We proposed that T17M rhodopsin may be misfolded and eliminated via ERAD, and chemical chaperone 4-phenylbutyrate may prevent ER stress induced by rhodopsin T17M.

In this study, we used a spontaneously arising retinal pigment epithelia (RPE) ARPE-19 cell line as the experimental model to investigate the role of ERAD in the clearance of T17M rhodopsin and the effects of 4-phenylbutyrate (4-PBA) on ER stress induced by rhodopsin T17M.

## Methods

### Cell culture and plasmid constructs

WT and T17M rhodopsin-myc constructs were described previously [10]. p97/VCP QQ-HA construct was described previously [12]. ARPE-19 cells were obtained from ATCC and cultured with DMEM supplemented with 10% FBS and penicillin-streptomycin (50 μg/ml) at 37°C in 5% CO_2_. 4-PBA (Sigma, St. Louis, MO, USA) was dissolved in filtered sterile water at 1 M stock concentration.

### Immunoblotting

Cells were lysed with RIPA sample buffer, the supernatant was collected and protein concentration was determined using a Pierce protein assay kit (Thermo Scientific). 30 μg proteins were separated on SDS-PAGE gel and transferred to PVDF membrane (Millipore). The membrane was incubated for 1 h in a blocking solution (5% dry milk in 0.1% triton X-100/PBS buffer) followed by incubation with appropriate primary antibodies in a blocking solution. After being washed in 0.1% triton X-100/PBS buffer, the membrane was incubated in appropriate secondary antibodies for 1 h and visualized via an enhanced chemiluminescence kit (GE Health) according to the manufacturer’s instruction. Antibodies were as follows: actin, HA antibodies (Abcam), Myc, Erasin, GRP78, GRP94, CHOP, peIF-2α, eIF-2α, and active ATF-6α antibodies (Cell Signaling), GFP antibody (Invitrogen).

### Analysis of protein turnover

ARPE-19 cells were transfected with erasin SMARTpool siRNAs (Dharmacon) using Dharmafect 1 reagent (Thermo Fisher Scientific) according to the manufacturer’s instructions. 48 h later, cells were transfected with Myc-tagged rhpdopsin using lipofection 2000 (Invitrogen). After 24 h, cells were treated with 50 μg/ml cycloheximide (Sigma) and collected at the indicated time points. For turnover experiments with p97/VCP QQ-HA, DNA construct was co-transfected with Myc-tagged rhpdopsin plasmid DNA followed by the inhibition of protein synthesis with cycloheximide. Equal amounts of protein in lysates from the different time points were separated by SDS–PAGE and immunoblotted.

### RNA extraction, RT-PCR and XBP1 splicing assay

Total RNA was extracted from cells with TRIzol (Invitrogen) and cDNA was synthesized with a High Capacity cDNA Reverse Transcription kit (Applied Biosystems). Primers encompassing the spliced sequences in XBP1 mRNA (5′-ACACGCTTGGGAATGGACAC-3′ and 5′-CCATGGGAAGATGTTCTGGG-3′) and loading control Actin (5’-GCGAGAAGATGACCCAGATC-3’, and 5’-CCAGTGGTACGGCCAGAGG-3’) were used for PCR, and products were separated by electrophoresis through a 2.5% agarose gel and visualized by ethidium bromide staining.

### Apoptosis detection

ARPE-19 cells were transfected with myc-tagged wild-type or T17M mutant constructs. Twenty hours after transfection, apoptosis was detected by Annexin V-FITC Apoptosis Detection Kit (Sigma). Briefly, cells were collected and washed twice with PBS. Then cells were resuspended in 500 μL Binding Buffer, and incubated with the addition of 5 μL Annex V-FITC and 10 μL propidium iodide at 37°C for 15 min in the dark. Next the stained samples were subjected to flow cytometry analysis to detect the apoptosis rate.

### Determination of cellular 26S proteasome activity

A stable cell line overexpressing GFP^U^ in ARPE-19 cells was established as a cellular model to test 26S proteasome activity after G418 selection. The reporter gene consisted of a short tag CL1 fused to the C-terminus of GFP. CL1, encoding a fragment of amino acids (ACKNWFSSLSHFVIHL), was shown to be a degradation substrate for ubiquitin-proteasome system [13]. The expression level of GFP^U^ after the transfection of rhodopsin T17M or WT was determined by immunoblotting.

### Statistical analysis

All experiments were repeated at least three times. Results were presented as means ± standard deviation (SD). Statistical significance of differences was evaluated with one way ANOVA followed by the Tukey test and Dunnett’s test. p < 0.05 was considered as statistical significance. GraphPad Prism 5 software was used for the analysis.

## Results

### Rhodopsin T17M mutant protein is ubiquitinated

**Figure 1:**
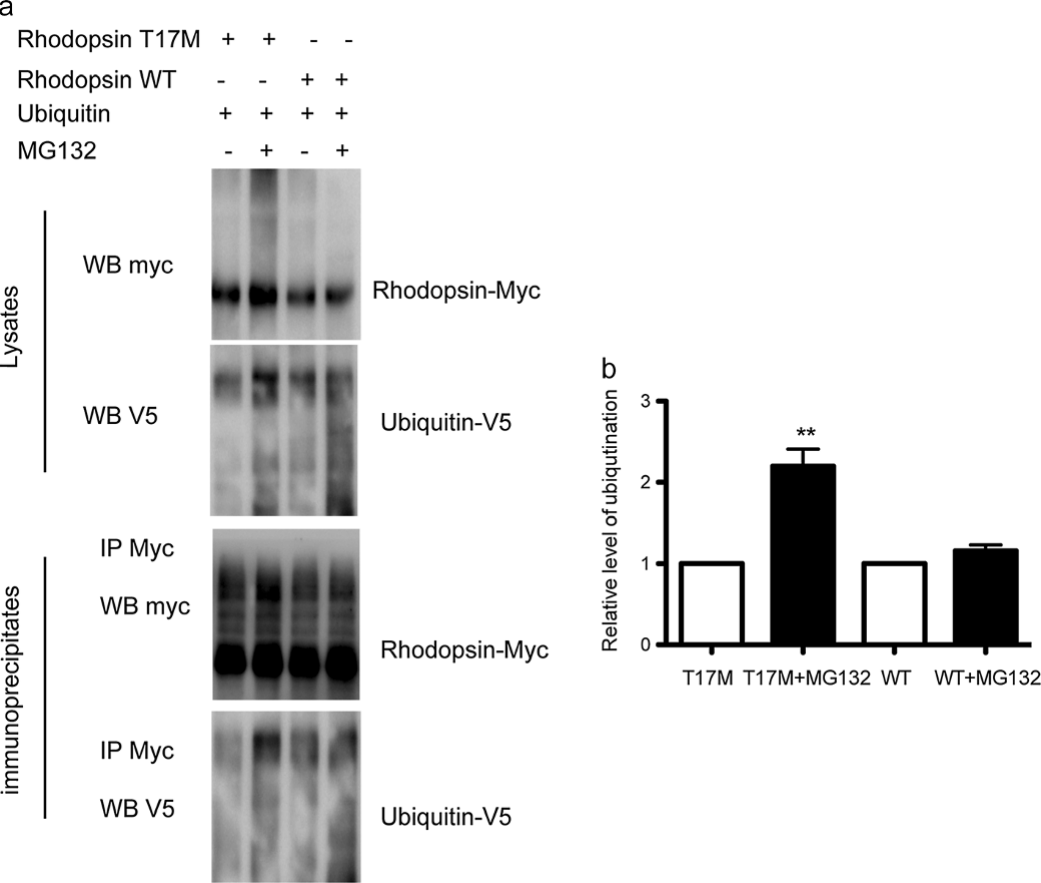
**Ubiquitination of myc-tagged rhodopsin T17M. (a)**. Upper: ARPE-19 cells were transfected with myc-tagged rhodopsin T17M or WT expression constructs. At 24 h post-transfection, the cells were treated with MG132 (10 μM) or DMSO for 6 h. The protein was detected by immunoblotting. Lower: Immunoprecipitation of transfected myc-tagged rhodopsin T17M or WT from ARPE-19 cells after MG132 treatment. The immunoprecipitates were immunoblotted for myc and ubiquitin-V5 **(b)**. Quantification of the relative level of ubiquitination. Data were presented as mean ± S.D. ** p < 0.01: compared to T17M cells.

Proteins that are terminally misfolded in the ER may be degraded by the proteasome via ERAD. Typically, such proteins are unstable and show a reduced half-life. Because ERAD substrates are typically ubiquitinated [14], we examined whether rhodopsin T17M protein is modified by ubiquitination. We immunoprecipitated Myc-tagged wild-type and T17M mutant rhodopsin proteins from ARPE-19 cells using myc antibody and probed the immunoprecipitates. As shown in Figure 1, considerably more rhodopsin T17M protein was observed in cell treated with MG132 than in untreated cells, confirming that proteasome inhibition leads to the stabilization of rhodopsin T17M. After immunoprecipitation and normalization, we observed a smear on the upper portions of the gels by using ubiquitin antibody, especially after MG312 treatment, indicating that rhodopsin T17M protein is modified by ubiquitination.

### Rhodopsin T17M mutant protein is degraded via ERAD pathway

**Figure 2:**
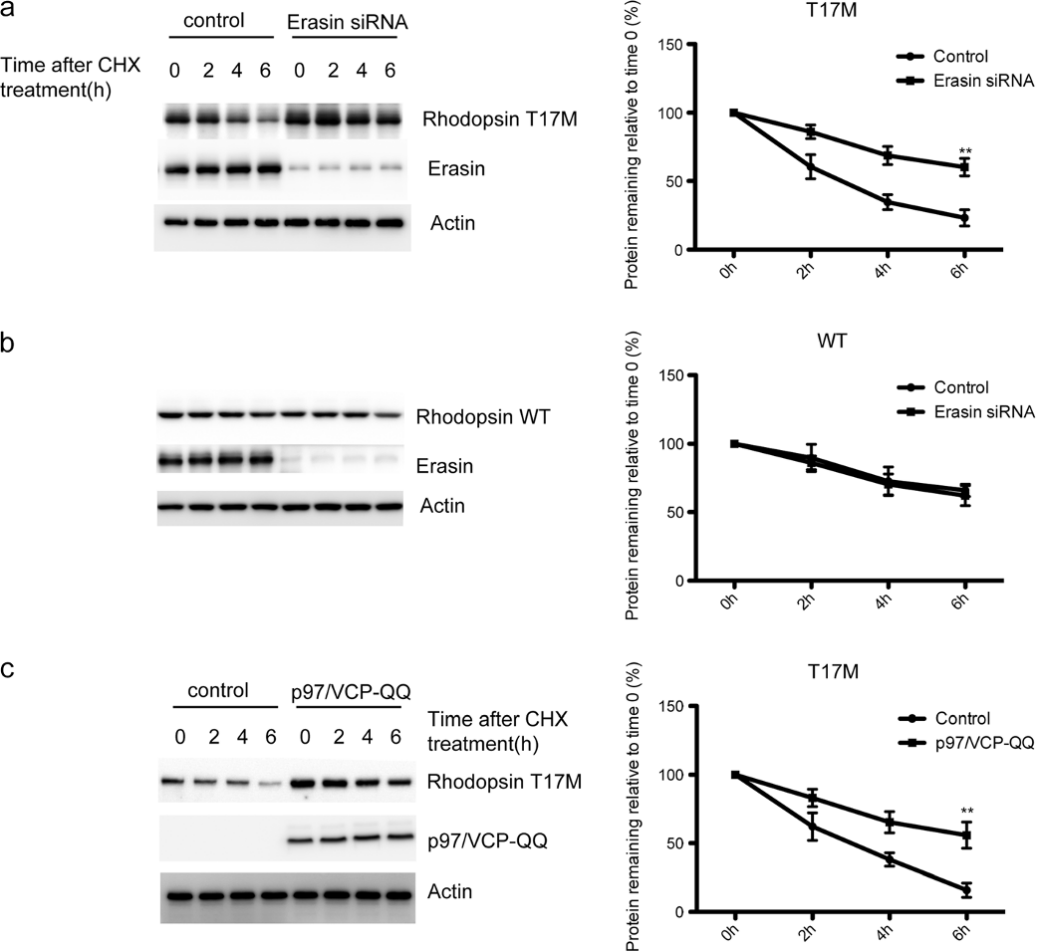
**Knockdown of erasin and overexpression of p97/VCP-QQ inhibit rhodopsin turnover.** ARPE-19 cells were transfected with myc-tagged rhodopsin T17M **(a)** or WT **(b)** expression constructs 48 h after transfection with erasin siRNAs. At 72 h post-knockdown, cells were treated with cycloheximide for the indicated time points and the proteins were detected by immunoblotting. ARPE-19 cells were cotransfected with HA-tagged p97/VCP-QQ and rhodopsin T17M **(c)**. 36 h after co-transfection, cells were treated with cycloheximide for the indicated time points and the proteins were detected by immunoblotting. Quantification of relative level of remained WT or T17M rhodopsin was shown in right panels. Data were presented as mean ± S.D. **p < 0.01 compared to Control.

To examine whether rhodopsin T17M mutant protein is eliminated by ERAD pathway, we disrupted two components involved in ERAD pathway to see how this would affect the turnover of rhodopsin T17M protein. The first protein we targeted was erasin. Erasin promoted ERAD. Overexpression of erasin enhanced the degradation of ERAD substrates, whereas siRNA-mediated reduction of erasin expression almost completely blocked ERAD [15]. We transfected ARPE-19 cells with myc-tagged WT or rhodopsin T17M after siRNA-mediated knockdown of erasin, and assessed protein turnover by cycloheximide chase analysis. The results showed that rhodopsin T17M was readily degraded with a half-life of around 4 h, whereas it was stable over the 6 h chase period in erasin knockdown cells. Disruption of erasin also induced increased rhodopsin T17M at 0 h cycloheximide treatment (Figure 2a). In contrast, knockdown of erasin had no significant effect on the turnover of rhodopsin WT protein (Figure 2b).

We next examined p97/VCP, which is essential for the dislocation of proteins from ER during ERAD [16]. We utilized a dominant-negative ATPase deficient p97/VCP-QQ mutant, which has been shown to slow the degradation of ERAD substrates. Accordingly, we cotransfected ARPE-19 cells with HA-tagged p97/VCP-QQ and myc-tagged WT or rhodopsin T17M, and assessed protein turnover by cycloheximide chase analysis. The results showed that the amounts of rhodopsin T17M was much higher in p97/VCP-QQ overexpressing cells than in control cells, indicating that p97/VCP-QQ inhibited the turnover of rhodopsin T17M (Figure 2c). Taken together, these results strongly indicate that rhodopsin T17M mutant protein is degraded via ERAD pathway.

### Rhodopsin T17M mutant protein has no effect on ubiquitin-proteasome system

**Figure 3:**
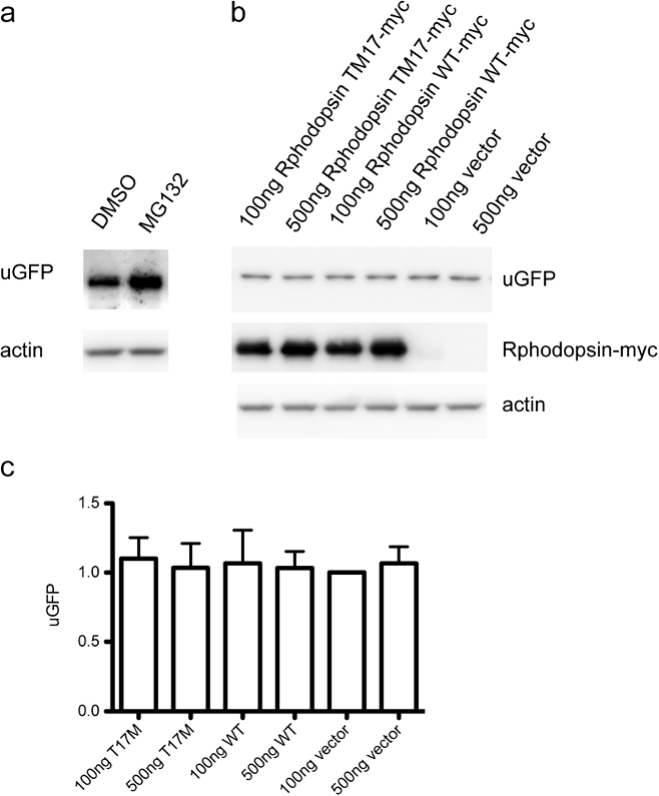
**Effect of rhodopsin T17M on ubiquitin-proteasome system activity. (a)** ARPE-19 cells stably expressing GFP^U^ were incubated with or without MG132 for 2 h to block the proteasome. **(b)** ARPE-19 cells stably expressing GFP^U^ were transiently transfected with rhodopsin T17M or WT. Lysates were immunoblotted with the antibody to GFP or myc. **(c)** Quantification of relative GFP level shown in **(b)**. Data were presented as mean ± S.D.

Ubiquitin-proteasome system (UPS) dysfunction is an important pathogenic factor in neurodegeneration diseases. The most common rhodopsin mutation P23H forms aggregates in ER and impairs UPS [17]. To investigate the effect of rhodopsin T17M on proteasome, ARPE-19 cells with stable expression of a GFP-conjugated proteasome degradation signal GFP^U^ was used [13]. The GFP^U^ reporter consists of a short degron, CL1, fused to the C-terminus of GFP. The product of GFP^U^ was continuously degraded and kept at a very low level under normal conditions. A decline of GFP^U^ level reflects ubiquitin-proteasome system activity, whereas an increase of level indicates that UPS activity is reduced or impaired. We have established a GFP^U^ stable expression ARPE-19 cell line using G418 selection. The GFP^U^ protein was significantly increased when proteasome was blocked by MG132, a proteasome inhibitor (Figure 3a). Then, ARPE-19 cells were transiently transfected with WT or rhodopsin T17M, and GFP^U^ level was detected. The results showed that there were no significant differences in GFPU level between WT and rhpdopsin T17M (Figure 3), indicating that Rhodopsin T17M mutant protein has no effect on UPS activity.

### Rhodopsin T17M mutant protein induces unfolded protein response

**Figure 4:**
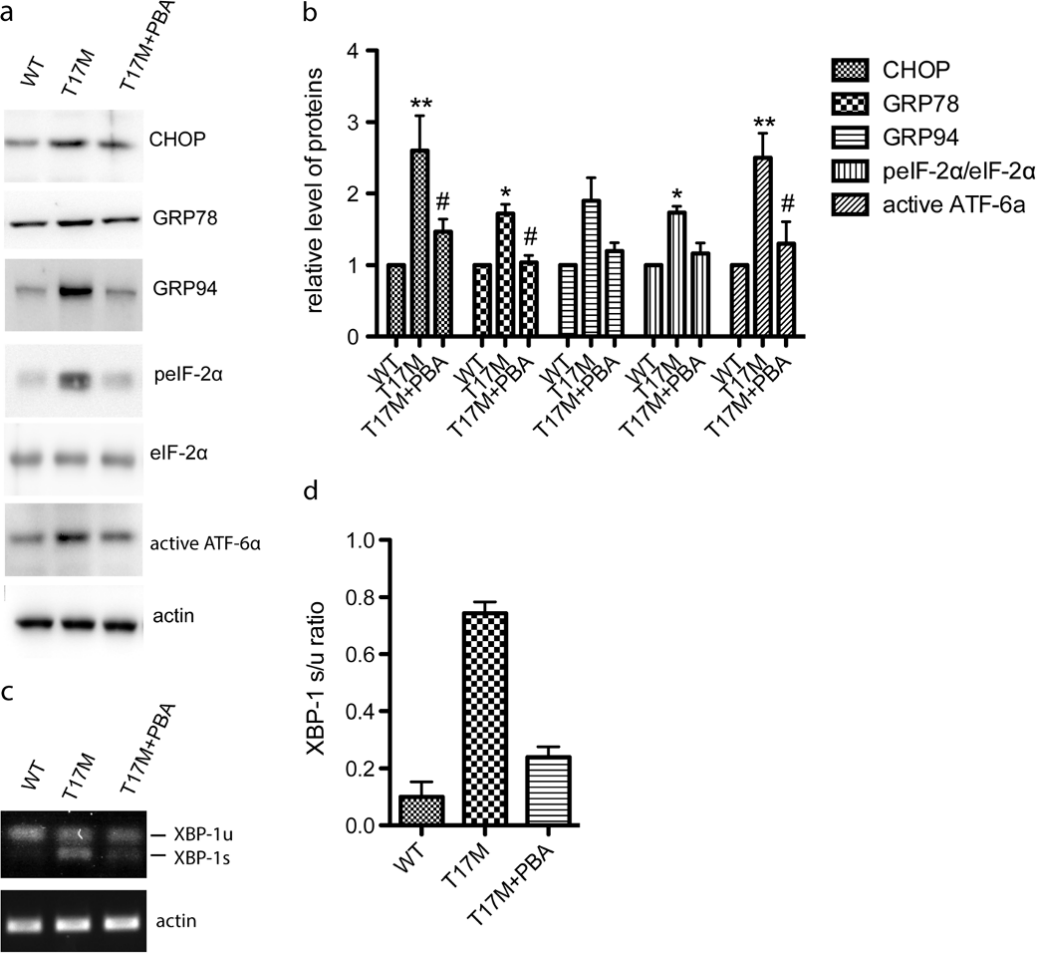
**4-PBA alleviates UPR and ER stress induced by rhodopsin T17M mutant.** ARPE-19 cells were transfected with myc-tagged rhodopsin T17M or WT expression constructs and treated with 5 mM 4-PBA. After 24 h, the indicated proteins **(a)** and splicing of XBP-1**(c)** were detected by immunoblotting and RT-PCR, respectively. Quantification of the data in **(a)** and **(b)** were shown in **(c)** and **(d)**, respectively. Data were presented as mean ± S.D. *:T17M vs. Control; #: T17M + 4-PBA vs. T17M; **p < 0.01.; *p < 0.05; # p < 0.05.

The accumulation of misfolded proteins in ER can activate unfolded protein response (UPR). To determine whether misfolding of rhodopsin T17M would activate UPR, ARPE-19 cells were transiently transfected with WT or rhodopsin T17M expression vector, and the levels of ER chaperone and UPR-associated proteins such as GRP78, GRP94, CHOP, peIF-2α, eIF-2α, active ATF-6α were assessed by immunoblotting. Compared to control, rhodopsin T17M mutant increased the levels of these proteins (Figure 4).

### 4-phenylbutyrate prevents UPR induced by rhodopsin T17M

4-phenylbutyrate (4-PBA) is a small chemical chaperone that could reduce ER stress both *in vivo* and *in vitro*. Thus we used PBA to treat ARPE-19 cells overexpressing rhodopsin T17M. Western blot analysis showed that the levels of GRP78, GRP94, CHOP, peIF-2α, eIF-2α, and active ATF-6α were reduced after 4-PBA treatment (Figure 4a, b). We also detected the splicing of XBP-1 in ARPE-19 cells overexpressing rhodopsin T17M. The small size form of XBP-1(XBP-1 s) was observed in rhodopsin T17M overexpressing cells. As expected, 4-PBA reduces the splicing of XPB-1 induced by rhodopsin T17M (Figure 4c, d). Taken together, these data suggested that 4-PBA attenuates UPR signaling and ER stress induced by rhodopsin T17M in ARPE-19 cells.

### Phenylbutyric acid facilitates rhodopsin T17M degradation and inhibits apoptosis induced by rhodopsin T17M

**Figure 5:**
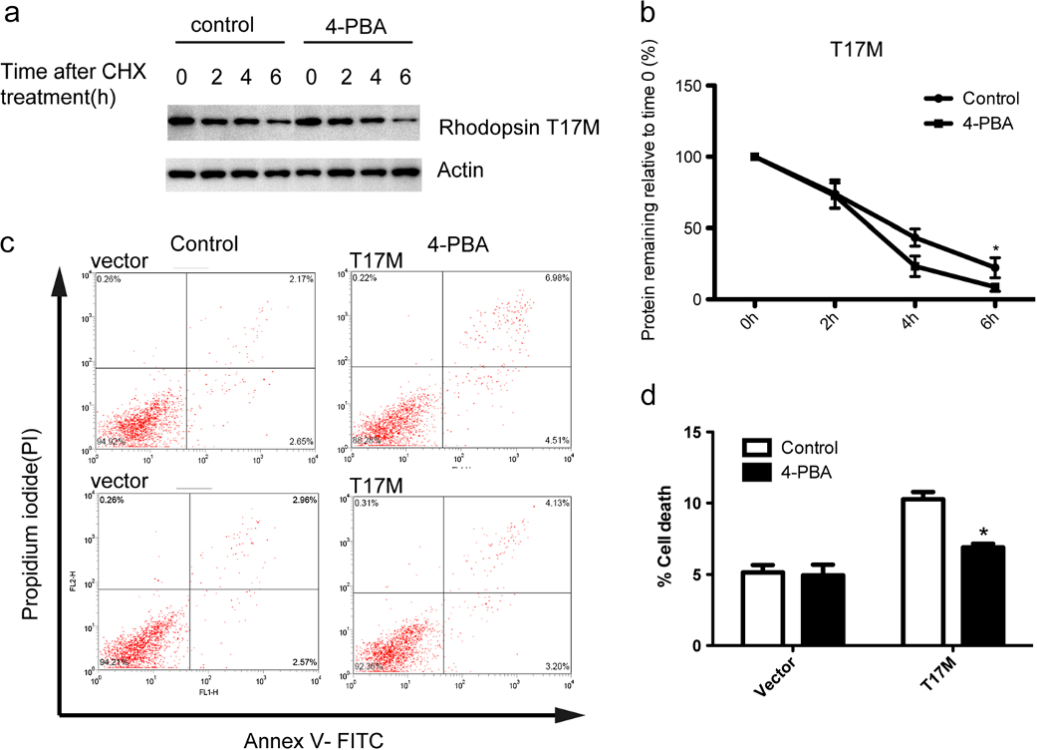
**4-PBA facilitates rhodopsin T17M degradation and inhibits apoptosis induced by rhodopsin T17M. (a)** ARPE-19 cells were transfected with myc-tagged rhodopsin T17M and untreated by 4-PBA (control) or treated by 5 mM 4-PBA. After 36 h, cells were treated with cycloheximide for the indicated time points and the proteins were detected by immunoblotting. **(b)** Quantification of the proteins shown in **(a)**. Data were presented as mean ± S.D. *p < 0.05. **(c)** ARPE-19 cells were stained with annexin V and PI. The apoptotic cells were annexin V-positive. **(d)** Quantification of apoptotic cells. Data were presented as mean ± S.D. *p < 0.05, compared with control.

To investigate whether 4-PBA has an effect on the turnover of rhodopsin T17M protein, we performed cycloheximide chase analysis. Upon 4-PBA treatment rhodopsin T17M had a short half-life of ~3 h, compared to ~4 h half-life in absence of 4-PBA (Figure 5a, b). However, we found that 4-PBA had no significant effect on intracellular localization of rhodopsin T17M in ARPE-19 cells (data not shown). Rhodopsin T17M protein is known to accumulate in ER and induce ER stress. Thus we wondered whether 4-PBA had protective effect on apoptosis induced by rhodopsin T17M. The results showed that the apoptosis rate was significantly higher in cells transfected with rhodopsin T17M expression vector than in cells transfected with empty vector. However, 4-PBA partially inhibited apoptosis induced by the overexpression of rhodopsin T17M (Figure 5c, d).

## Discussion

In this study, we found that rhodopsin T17M protein was ubiquitinated and the ubiquitination was increased following proteasome inhibitor treatment. Moreover, interference of ERAD either by overexpression of a dominant negative p97/VCP-QQ protein or by knockdown of erasin slowed the degradation of rhodopsin T17M mutant protein.

RPE is a monolayer of hexagonal cells separating the neural retina from the underlying choroidal vascular bed. RPE cells are essential for the development, survival, and physiological activity of photoreceptor cells [18]. Mutations in genes that are expressed in the RPE can lead to photoreceptor degeneration. On the other hand, mutations in genes expressed in photoreceptor cells can lead to degenerations of the RPE. Thus RPE and photoreceptors cells are closely linked [19]. It is found that rhodopsin is expressed in RPE cells [20]. The human retinal pigment epithelial cell line (ARPE-19), a transformed human retinal pigment epithelial cell line, was employed to investigate the effect of rhodopsin mutant on RPE degeneration. In addition, some mammalian cell lines, such as 293 s [21], HeLa [22] and COS [23] were used to investigate biological functions of rhodopsin involved in RP mechanism.

T17M is a type II mutant rhodopsin that traffics abnormally and forms pigment inefficiently [24]. Proper protein folding and processing is necessary to maintain cellular homeostasis. Protein misfolding could potentially not only affect function but also lead to protein aggregation and induce toxicity. Not surprisingly, cells have developed elaborate and complex systems to eliminate unwanted and potentially toxic proteins. One of the first quality control checkpoints is in the ER, where misfolded proteins are recognized and eliminated by ERAD. Although many mutations involved in human disease are thought to cause proteins to misfold, relatively few of them have been shown to be eliminated by ERAD. Here, we presented evidence that mutant rhodopsin protein linked to RP is degraded by ERAD.

Misfolded rhodopsin R32H is a substrate of the ERAD effector VCP, an ATP-dependent chaperone that extracts misfolded proteins from the ER and escorts them for proteasomal degradation [25]. Inactivation of VCP/ter94 suppresses retinal pathology caused by misfolded rhodopsin in Drosophila [26]. Co-expression of certain ERAD factors was sufficient to reduce Rh-1 protein levels and suppress ER stress reporter activation, indicating that ERAD acts as a protective mechanism against retinal degeneration in the Drosophila model for ADRP. These results suggest that manipulation of ERAD may serve as a powerful therapeutic strategy against a number of diseases associated with ER stress [27].

ER is responsible for the folding of secreted and membrane proteins in eukaryotic cells. Disruption of protein folding leads to ER stress. Chronic ER stress can cause cell death and is implicated in the pathogenesis of many human diseases. ER stress response is involved in retinal degeneration in T17M Rho mice and S334ter Rho rats [28]. Ablation of CHOP, selective activation of ATF6 or PERK, disruption of CDK5 and MEKK1 pathway, or the stimulation of ERAD may serve as a powerful therapeutic strategy against rhodopsin mutants induced RP and reverse severe retinal degeneration [29–32]. Chemical chaperones reduce ER stress and are proposed as therapeutic target for various diseases [33]. 4-PBA is a low molecular weight terminal aromatic substituted fatty acid approved for clinical use as an ammonia scavenger in children with urea cycle disorders [34]. Recent reports showed that 4-PBA has protective effects against ER stress-induced neuronal cell death [35, 36]. In this study our results showed that 4-PBA reduced ER stress induced by T17M rhodopsin. Thus 4-PBA is a promising agent to treat RP because it could prevent RP induced by T17M rhodopsin mutant. Although 4-PBA facilitates the degradation of T17M mutant and attenuates apoptosis induced by ER stress, it does not affect the location of T17M mutant. Further studies are necessary to investigate whether intracellular trafficking of immature or misfolded rhodopsin is a potential target for RP therapy.

## Conclusions

T17M rhodopsin is misfolded, ubiquitinated and eliminated by ER-associated degradation pathway. Chemical chaperone could attenuate UPR signaling and ER stress induced by T17M rhodopsin and has potential therapeutic significance for retinitis pigmentosa.
